# Mutations in *hmg1*, Challenging the Paradigm of Clinical Triazole Resistance in Aspergillus fumigatus

**DOI:** 10.1128/mBio.00437-19

**Published:** 2019-04-02

**Authors:** Jeffrey M. Rybak, Wenbo Ge, Nathan P. Wiederhold, Josie E. Parker, Steven L. Kelly, P. David Rogers, Jarrod R. Fortwendel

**Affiliations:** a Department of Clinical Pharmacy and Translational Science, University of Tennessee Health Science Center, Memphis, Tennessee, USA; b Fungus Testing Laboratory, Department of Pathology and Laboratory Medicine, University of Texas Health Science Center at San Antonio, San Antonio, Texas, USA; c Institute of Life Science, Swansea University Medical School, Swansea, Wales, United Kingdom; Duke University Medical Center

**Keywords:** *Aspergillus fumigatus*, HMG-CoA reductase, antifungal resistance, ergosterol, *hmg1*, triazole

## Abstract

Aspergillus fumigatus is the predominant pathogen of invasive aspergillosis, a disease state credited with over 200,000 life-threatening infections each year. The triazole class of antifungals are clinically essential to the treatment of invasive aspergillosis, both as frontline and as salvage therapy. Unfortunately, resistance to the triazoles among A. fumigatus isolates is now increasingly reported worldwide, and a large proportion of this resistance remains unexplained. In this work, we characterize the contributions of previously identified mechanisms of triazole resistance, including mutations in the sterol-demethylase-encoding gene *cyp51A*, overexpression of sterol-demethylase genes, and overexpression of the efflux pump-encoding gene *abcC*, among a large collection of highly triazole-resistant clinical A. fumigatus isolates. Upon revealing that these mechanisms alone cannot substantiate the majority of triazole resistance exhibited by this collection, we subsequently describe the identification and characterization of a novel genetic determinant of triazole resistance. Mutations in the 3-hydroxy-3-methyl-glutaryl-coenzyme A (HMG-CoA) reductase-encoding gene, *hmg1*, were identified in a majority of triazole-resistant clinical isolates in our collection. Introduction of three different *hmg1* mutations, predicted to encode residue alterations in the conserved sterol sensing domain of Hmg1, resulted in significantly increased resistance to the triazole class of agents. Additionally, correction of a *hmg1* mutation in a pan-triazole-resistant clinical isolate of A. fumigatus with a novel Cas9-ribonucleoprotein-mediated system was shown to restore clinical susceptibility to all triazole agents. Mutations in *hmg1* were also shown to lead to the accumulation of ergosterol precursors, such as eburicol, by sterol profiling, while not altering the expression of sterol-demethylase genes.

## Abstract (precis)

Aspergillus fumigatus is the predominant pathogen of invasive aspergillosis, a disease state credited with over 200,000 life-threatening infections annually. The triazole class of antifungals are clinically essential to the treatment of invasive aspergillosis. Unfortunately, resistance to the triazoles among A. fumigatus isolates is now increasingly reported worldwide. In this work, we challenge the current paradigm of clinical triazole resistance in A. fumigatus, by first demonstrating that previously characterized mechanisms of resistance have nominal impact on triazole susceptibility and subsequently identifying a novel mechanism of resistance with a profound impact on clinical triazole susceptibility. We demonstrate that mutations in the HMG-CoA reductase gene, *hmg1*, are common among resistant clinical isolates and that *hmg1* mutations confer resistance to all clinically available triazole antifungals.

## INTRODUCTION

Aspergillus fumigatus is the predominant pathogen isolated from patients with invasive aspergillosis, the most common human invasive mold infection. Invasive aspergillosis is responsible for more than 200,000 life-threatening infections each year and afflicts up to 10% of patients with acute leukemia as well as patients receiving stem cell or solid organ transplantation (1–3). Unfortunately, the mortality rates associated with invasive aspergillosis remain unacceptably high, often exceeding 50%, even when appropriately diagnosed and treated (3–6).

Contributing to the poor outcomes associated with the treatment of invasive aspergillosis is the relative paucity of clinically available antifungal agents with demonstrated clinical efficacy. Of the four major classes of antifungals, only the triazoles and amphotericin B are recommended as monotherapy for the treatment of aspergillosis, with the latter relegated to second-line or salvage treatment due to significant toxicities and the lack of oral formulations (7). The triazole class of antifungals therefore has proven essential to the treatment of invasive aspergillosis, comprising both frontline treatment options such as voriconazole and isavuconazole and salvage options such as posaconazole and itraconazole (7). These agents are generally accepted to exert fungicidal activity against *Aspergillus* through the competitive inhibition of sterol-demethylase, encoded by the genes *cyp51A* (Afu4g06890) and *cyp51B* (Afu7g03740) in A. fumigatus, which results in abrogation of the synthesis of ergosterol, the major fungal membrane sterol (8–11). However, the treatment of invasive aspergillosis has recently been further complicated by the global emergence of triazole-resistant disease. Over the past decade, triazole resistance among clinical isolates of A. fumigatus has been increasingly reported on six continents, with resistance rates exceeding 20% now being reported in some studies (12–15).

Considerable research has led to the identification of three distinct molecular pathways to clinical triazole resistance in A. fumigatus. These three paths largely mirror the established mechanisms of resistance previously identified in other pathogenic fungi such as Candida albicans: (i) mutations in the sterol-demethylase gene *cyp51A*, (ii) overexpression of the sterol-demethylase gene *cyp51A*, and (iii) overexpression of the efflux pump-encoding gene *abcC* (16–25). However, it remains unknown to what extent these mechanisms explain the degree of triazole resistance observed among clinical isolates of A. fumigatus, and resistant clinical isolates exhibiting none of these three mechanisms have been repeatedly identified (22). Thus, the molecular mechanisms underpinning much of the clinical resistance to this essential class of anti-*Aspergillus* agents remain unexplained, limiting the discovery of therapeutic strategies to overcome triazole-resistant aspergillosis.

In this work, we perform a comprehensive characterization of the direct contributions of previously identified mechanisms of triazole resistance in a large collection of triazole-resistant clinical isolates of A. fumigatus. We correct *cyp51A* mutations in 10 different triazole-resistant clinical isolates using a novel Cas9-mediated transformation system to delineate the direct impact these mutations have on clinical triazole susceptibility, and we assess the potential impact of overexpression of *cyp51A*, *cyp51B*, and *abcC* on triazole susceptibility in a collection of 21 triazole-resistant clinical isolates. We subsequently demonstrate that these mechanisms alone fail to explain the resistance observed in this collection. We further describe here, for the first time, a novel mechanism of clinical triazole resistance which was found to be present in a majority of triazole-resistant clinical isolates in our collection. Mutations in the 3-hydroxy-3-methyl-glutaryl-coenzyme A (HMG-CoA) reductase-encoding gene, *hmg1* (Afu2g03700), were found to result in dramatically increased resistance to all clinically available triazole agents. Additionally, the restoration of *hmg1* to the wild-type sequence in a clinical A. fumigatus isolate exhibiting high-level pan-triazole resistance was found to restore clinical susceptibility to the triazole class. We demonstrate that mutations in *hmg1* result in sustained or even increased ergosterol production as well as accumulation of multiple ergosterol precursors, including eburicol, the substrate of the sterol-demethylase enzymes, which the triazole antifungals competitively inhibit.

## RESULTS

### Mutations in sterol-demethylase alone poorly explain clinical triazole resistance.

Mutations in the sterol-demethylase gene *cyp51A* are the most frequently identified mechanism of triazole resistance in A. fumigatus (6, 15, 26). We sought to delineate the proportion of triazole resistance observed in individual clinical isolates explained by this mechanism. To accomplish this, a collection of 26 clinical isolates of A. fumigatus originating from the United States was obtained from the Fungus Testing Laboratory at the University of Texas Health Science Center at San Antonio, including 21 multitriazole-resistant isolates with previously reported clinical origins and characterized *cyp51A* genotypes and 5 triazole-susceptible control isolates (15). High-quality genomic DNA was extracted from each of the 26 isolates, whole-genome sequencing was performed targeting a read depth of approximately 100 reads per base aligned to the Af293 A. fumigatus reference genome assembly, and a total of 48,552 SNPs and insertions or deletions (indels) unique to triazole-resistant isolates were identified within 8,669 genes with annotations available. Of the 21 triazole-resistant isolates in this collection, 16 were identified as possessing peptide-sequence-altering *cyp51A* mutations unique to resistant isolates. Conversely, no unique *cyp51B* mutations were identified. We selected 10 of these 16 isolates with *cyp51A* mutations, which also exhibited resistance to all clinically available triazole agents, for correction of the *cyp51A* sequence to that of the wild-type consensus sequence. These isolates included examples of previously characterized *cyp51A* mutations (TR_34_/L98H, TR_46_/Y121F/T289A, and G448S), mutations at residues where alternative mutations have previously been characterized (G138C and F219S), and mutations never before characterized to our knowledge (M263I and I367F) (Table 1) (17, 24, 25, 27, 28).

**TABLE 1:** Triazole MICs of clinical isolates and derivative *cyp51A*^WT^ strains^*a*^

Parent clinical isolate or derivative *cyp51A*^WT^ strain	*cyp51A* genotype	MIC (mg/liter) of triazole:		
		Voriconazole	Itraconazole	Posaconazole
DI15-117	F219S	**2**	**≥32**	**2**
DI15-117 *cyp51A*^WT^	WT	**4**	**≥32**	**4**
DI15-100	M263I	**8**	**≥32**	**1**
DI15-100 *cyp51A*^WT^	WT	**8**	**≥32**	**0.5**
DI15-116	TR_34_/L98H	**8**	**≥32**	**1**
DI15-116 *cyp51A*^WT^	WT	1	0.25	0.125
DI15-102	TR_34_/L98H	**8**	**≥32**	**2**
DI15-102 *cyp51A*^WT^	WT	**4**	**≥32**	**0.5**
DI15-108	G138C	**4**	**≥32**	**1**
DI15-108 *cyp51A*^WT^	WT	**4**	**≥32**	0.125
DI15-95	M263I	**16**	**≥32**	**2**
DI15-95 *cyp51A*^WT^	WT	**16**	**≥32**	**1**
DI15-98	I367F	**16**	**16**	**2**
DI15-98 *cyp51A*^WT^	WT	**16**	**≥32**	**1**
DI15-96	TR_46_/Y121F/T289A	**≥32**	**4**	**1**
DI15-96 *cyp51A*^WT^	WT	**≥32**	0.5	0.25
DI15-106	TR_46_/Y121F/T289A	**≥32**	**4**	**1**
DI15-106 *cyp51A*^WT^	WT	**8**	**2**	**0.5**
DI15-120	G448S	**≥32**	**≥32**	**1**
DI15-120 *cyp51A*^WT^	WT	**≥32**	**≥32**	**1**

a MICs shown in bold are greater than epidemiologic cutoff values as published by the Clinical Laboratory and Standards Institute (1 mg/liter for voriconazole and itraconazole; 0.25 mg/liter for posaconazole). WT, *cyp51A* sequence matches the Af293 wild-type consensus sequence.

In order to test the direct contribution of these mutations to clinical triazole resistance in these isolates, the wild-type *cyp51A* promoter region and open reading frame from the triazole-susceptible reference strain Af293 were first cloned upstream of the hygromycin resistance cassette in plasmid pCR-HygR (29). Fifteen hundred bases of the *cyp51A* terminator was then cloned downstream of the hygromycin resistance cassette, yielding the plasmid pCyp51A-HygR. Then, the *cyp51A*^WT^ transformation repair template was created by PCR using primers which amplified the wild-type *cyp51A* allele (including 500 bases of promoter sequence), the hygromycin resistance cassette, and 500 bases of the *cyp51A* terminator. Importantly, this repair template extends approximately 200 bases upstream of the promoter region where characterized regulatory elements and tandem repeat sequences have been identified (21). Protoplasts of each of the 10 triazole-resistant clinical isolates, as well as the well-characterized triazole-susceptible laboratory strain *akuB*^KU80^ (CEA17 *ΔakuB*^KU80^), which has a wild-type *cyp51A* allele, were then transformed with Cas9-ribonucleoprotein (RNP) complexes, consisting of Cas9 protein and CRISPR RNA (crRNA) and trans-activating CRISPR RNA (trRNA) duplexes targeting sequences immediately upstream and downstream of the *cyp51A* locus. The resulting transformants were selected on *Aspergillus* minimal medium containing hygromycin as previously described (29, 30). Integration of the repair template at the native *cyp51A* locus was confirmed by PCR, and acquisition of the *cyp51A*^WT^ sequence was verified by Sanger sequencing.

Clinical susceptibility testing was performed for each of the 10 triazole-resistant clinical isolates and the corresponding *cyp51A*^WT^ derivative strains for voriconazole, itraconazole, and posaconazole according to the Clinical and Laboratory Standards Institute M38-A2 standards (Table 1) (31). All of the resistant clinical isolates exhibited triazole MICs consistent with those previously reported for these isolates under clinical susceptibility testing conditions, and all MICs for each agent tested were above epidemiologic cutoff values previously reported by CLSI (1 mg/liter for voriconazole and itraconazole and 0.25 mg/liter for posaconazole) (15, 32). Among the *cyp51A*^WT^ strains, only one strain (DI15-116 *cyp51A*^WT^) was observed to have full restoration of wild-type triazole susceptibility, and two strains (DI15-96 *cyp51A*^WT^ and DI15-108 *cyp51A*^WT^) were observed to have restoration of wild-type susceptibility to at least one triazole. Additionally, two strains (DI15-102 *cyp51A*^WT^ and DI15-106 *cyp51A*^WT^) were observed to have at least one triazole MIC decrease by more than 1 dilution. Five of the 10 *cyp51A*^WT^ derivative strains exhibited no change in susceptibility to any triazole agent tested. Importantly, replacement of the native wild-type *cyp51A* allele with the *cyp51A*^WT^ cassette in the *akuB*^KU80^ background had no effect on triazole MIC. Taken together, these data demonstrate that while mutations in *cyp51A* are common among triazole-resistant clinical isolates of A. fumigatus, and a number of these mutations have been demonstrated to increase triazole MICs when introduced into a triazole-susceptible background, mutations in *cyp51A* alone poorly explain the high level of triazole resistance observed in this collection of clinical isolates.

### Analysis of the expression of the sterol-demethylase genes *cyp51A* and *cyp51B* among triazole-resistant clinical isolates.

Triazole-resistant clinical isolates of A. fumigatus exhibiting overexpression of *cyp51A* and *cyp51B*, relative to susceptible comparator isolates, have previously been reported (22, 33, 34). Additionally, both the formation of tandem repeats (TR_34_, TR_46_, and TR_53_) in the promoter region of *cyp51A* and a mutation (encoding the amino acid substitution P88L) in *hapE*, a CCAAT-binding complex protein-encoding gene which participates in the negative regulation *cyp51A* expression, have been shown to increase both *cyp51A* expression and triazole MICs (20, 21, 24, 25). Following the observation that mutations in *cyp51A* alone do not explain the degree of triazole resistance observed in this collection of clinical isolates, we sought to evaluate the potential contributions of sterol-demethylase gene overexpression to clinical triazole resistance. To accomplish this, each of the 26 clinical isolates in our collection was grown overnight at 37°C in *Aspergillus* minimal medium in biological triplicate, and RNA was extracted from mature hyphae following liquid nitrogen crush as previously described (35). Reverse transcription-quantitative-PCR (RT-qPCR) was then utilized to quantify the relative expression of both *cyp51A* and *cyp51B* in each of the 21 triazole-resistant clinical isolates compared to the expression level observed among the 5 triazole-susceptible control isolates.

**FIG 1:**
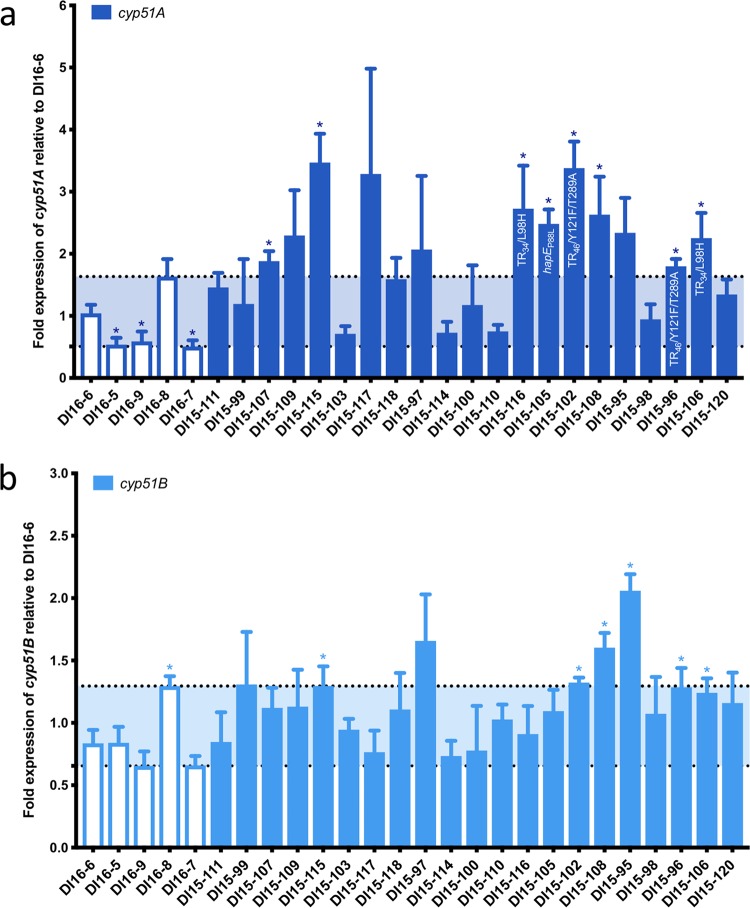
Relative expression of *cyp51A* and *cyp51B* among triazole-resistant clinical isolates. (a) Expression of *cyp51A* in clinical isolates relative to triazole-susceptible isolate DI16-6; identified mutations known to contribute to *cyp51A* overexpression are shown on the corresponding bar. (b) Expression of *cyp51B* in clinical isolates relative to triazole-susceptible isolate DI16-6; triazole-susceptible isolates are shown as open bars, and triazole-resistant isolates are shown as filled bars. Triazole-resistant isolates shown in order of escalating voriconazole MICs from left to right. Horizontal dotted lines denote highest and lowest expression levels observed among the 5 triazole-susceptible isolates. Comparisons with the susceptible control isolate DI16-6 with statistically significant (*P* < 0.05) results are noted with an asterisk.

The expression of *cyp51A* and *cyp51B* in all clinical isolates was compared to the triazole-susceptible isolate DI16-6 (voriconazole MIC, 0.5 mg/liter). Among triazole-susceptible control isolates, the expression of *cyp51A* ranged from 0.5- to 1.6-fold that of isolate DI16-6 (Fig. 1a), with isolates DI16-5, DI16-7, and DI16-9 exhibiting statistically significant lower expression of *cyp51A*. The expression of *cyp51A* among triazole-resistant clinical isolates ranged from 0.7- to 3.5-fold that of the susceptible comparator isolate. Twelve of the 21 triazole-resistant isolates were observed to have *cyp51A* expression that exceeded the highest expression level observed among triazole-susceptible isolates. Of these 12 isolates, 8 were found to have *cyp51A* expression levels statistically greater than the susceptible comparator isolate. This included 2 isolates (DI15-102 and DI15-116) which possess the TR_34_/L98H *cyp51A* mutation, 2 isolates (DI15-96 and DI15-106) which possess the TR_46_/Y121F/T289A *cyp51A* mutation, and one isolate (DI15-105) which possesses the previously characterized P88L-encoding mutation in *hapE.* However, no previously characterized mutations known to contribute to *cyp51A* overexpression were identified in the remaining 3 isolates. The expression of *cyp51B* among the triazole-susceptible control isolates ranged from 0.7- to 1.3-fold that of isolate DI16-6 (Fig. 1b), with isolate DI16-8 exhibing statistically greater expression of *cyp51B* (1.3-fold). The expression of *cyp51B* among triazole-resistant clinical isolates ranged from 0.7- to 2.1-fold that of the susceptible comparator isolate, with 4 isolates (DI15-95, DI15-97, DI15-102, and DI15-108) exhibiting expression of *cyp51B* exceeding that of the highest expression level observed among triazole-susceptible isolates, 3 of which (DI15-95, DI15-102, and DI15-108) were statistically significant.

### Constitutive overexpression of *cyp51B* decreases triazole susceptibility.

**FIG 2:**
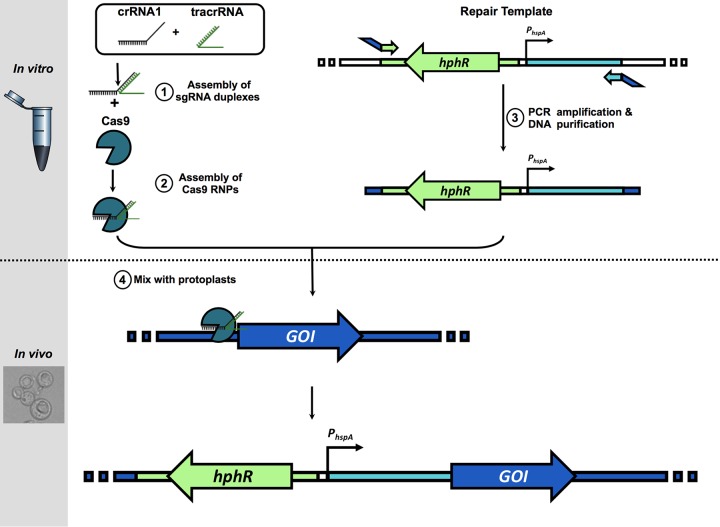
Schematic of Cas9-RNP-mediated promoter replacement system. Constitutive overexpression repair templates were amplified from pJMR2 using primers incorporating microhomology targeting genes of interest (GOI) and then mixed with Cas9-RNP targeting immediately upstream of the start codon of the GOI, and protoplasts were created from the strain *akuB*^KU80^.

Overexpression of *cyp51A* has previously been shown to contribute to triazole resistance, but it remains unknown if overexpression of the paralogous gene, *cyp51B,* has a similar effect on triazole susceptibility. We sought to employ a novel Cas9-RNP mediated promoter replacement system to delineate the direct impact of constitutive overexpression of *cyp51B* on triazole MIC. To accomplish this, the previously characterized, strong, constitutive promoter from the heat shock protein-encoding gene, *hspA* (Afu1g07440), was cloned upstream of the hygromycin resistance cassette in the plasmid pCR-HygR (36). A transformation repair template for the *cyp51B* promoter replacement was then constructed by amplifying the *hspA* promoter and hygromycin resistance cassette from the resulting plasmid (pJMR2) with primers that introduced approximately 50 bases of homology for the 3′ end of the native promoter region and the 5′ end of the *cyp51B* open reading frame. Protoplasts of the well-characterized triazole-susceptible laboratory strain *akuB*^KU80^ were then transformed with Cas9-ribonucleoprotein (RNP) complexes, consisting of Cas9 protein and crRNA-trRNA duplexes targeting sequences immediately upstream of the *cyp51B* start codon, yielding the strain *akuB*^KU80^_*P_hspA_cyp51B* (Fig. 2) (37). As a comparator, the same methodology was employed to create the constitutive *cyp51A-*overexpressing strain *akuB*^KU80^_*P_hspA_cyp51A*.

**FIG 3:**
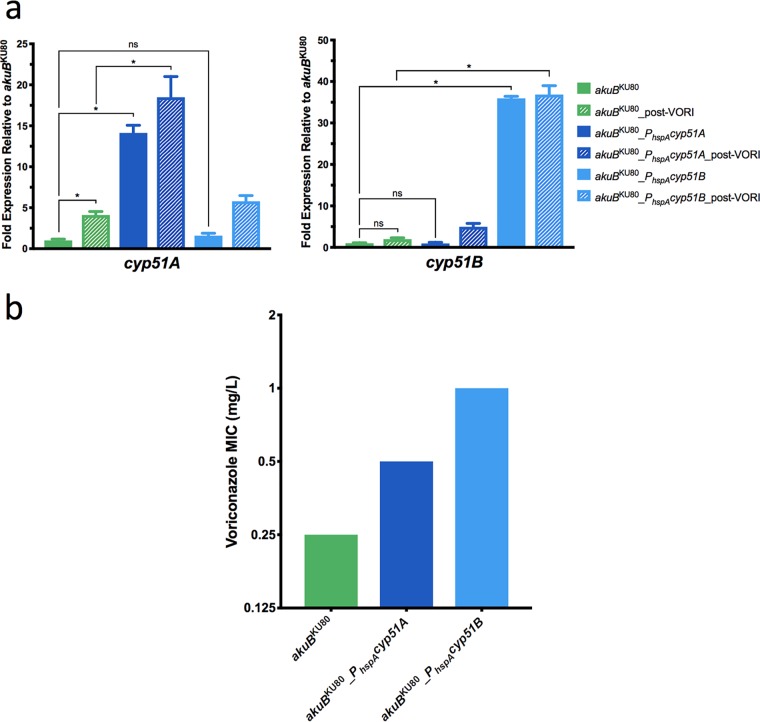
Relative expression of *cyp51A* and *cyp51B* and voriconazole MICs in promoter replacement strains *akuB*^KU80^_*P_hspA_cyp51A* and *akuB*^KU80^_*P_hspA_cyp51B*. (a) The expression of *cyp51A* (left) and *cyp51B* (right) in *akuB*^KU80^, *akuB*^KU80^_*P_hspA_cyp51A*, and *akuB*^KU80^_*P_hspA_cyp51B* was determined by RT-qPCR following growth in *Aspergillus* minimal medium at 37°C both with voriconazole (0.125 mg/liter) treatment for 6 h and without voriconazole treatment. The expression level for each sample is shown relative to that of the respective gene in *akuB*^KU80^ without voriconazole treatment. (b) Voriconazole MICs for *akuB*^KU80^, *akuB*^KU80^_*P_hspA_cyp51A*, and *akuB*^KU80^_*P_hspA_cyp51B*. Comparisons with statistically significant (*P* < 0.05) results are noted with an asterisk, while those that are not are noted with “ns.”

The relative expression of both *cyp51A* and *cyp51B* in the strains *akuB*^KU80^_*P_hspA_cyp51A* and *akuB*^KU80^_*P_hspA_cyp51B* was then compared to the parental *akuB*^KU80^ strain both under normal growth conditions and following voriconazole treatment. Conidia from each strain were grown in *Aspergillus* minimal medium at 37°C until germination and then were transferred to fresh *Aspergillus* minimal medium containing either no voriconazole or 0.125 mg/liter of voriconazole (half of the MIC of the parental *akuB*^KU80^ strain) for an additional 6 h at 37°C. RNA was extracted from each sample as previously described, and RT-qPCR was performed. The expression of *cyp51B* in *akuB*^KU80^ was 2-fold higher after treatment with voriconazole than under untreated conditions (Fig. 3a). Expression of *cyp51B* in the *akuB*^KU80^_*P_hspA_cyp51B* strain was 35.9-fold higher under untreated conditions and 36.9-fold higher following voriconazole exposure than the expression measured in the parental *akuB*^KU80^ under untreated conditions. This increased expression was statistically greater than in the parental strain under both treated and untreated conditions. The expression of *cyp51B* in the *akuB*^KU80^_*P_hspA_cyp51A* strain approximated that of *akuB*^KU80^ under both untreated and voriconazole-treated conditions. The expression of *cyp51A* in *akuB*^KU80^ was 4.1-fold higher following treatment with voriconazole than under untreated conditions. Expression of *cyp51A* in the *akuB*^KU80^_*P_hspA_cyp51A* strain was 14.1-fold higher under untreated conditions, and 18.5-fold higher following voriconazole exposure, than the expression measured in the parental *akuB*^KU80^ under untreated conditions. This increased expression was statistically greater than in the parental strain under both treated and untreated conditions. The expression of *cyp51A* in the *akuB*^KU80^_*P_hspA_cyp51B* strain was similar to that of *akuB*^KU80^ under both untreated and voriconazole-treated conditions.

Upon confirmation of the constitutive overexpression of *cyp51A* and *cyp51B* in the *akuB*^KU80^_*P_hspA_cyp51A* and *akuB*^KU80^_*P_hspA_cyp51B* strains, respectively, voriconazole MICs were determined in triplicate according to CLSI M38-A2 standards (31). The parental *akuB*^KU80^ strain exhibited a voriconazole MIC of 0.25 mg/liter. The *akuB*^KU80^_*P_hspA_cyp51A* strain was observed to have a 2-fold increase in voriconazole MIC (0.5 mg/liter), which is similar to the decrease in susceptibility previously reported when expression of this gene is increased as a result of either the formation of tandem repeats in the *cyp51A* promoter or a mutation in *hapE* (Fig. 3b) (20, 21, 24, 25). Interestingly, the voriconazole MIC of the *akuB*^KU80^_*P_hspA_cyp51B* strain was 1 mg/liter, 4-fold higher than the parental isolate. Taken together, these results demonstrate that overexpression of either sterol-demethylase gene can decrease triazole susceptibility. However, even when the expression of either of these genes increased by as much as 36.9-fold, the MIC did not exceed the epidemiological cutoff value for voriconazole reported by CLSI (1 mg/liter) (32).

### Analysis of the expression of the efflux pump-encoding gene *abcC* among triazole-resistant clinical isolates.

In addition to sterol-demethylase-mediated mechanisms of triazole resistance, increased constitutive expression of the ATP-binding cassette efflux pump-encoding gene, *abcC* (Afu1g14330; also known as *abcB*, *cdr1B*, and *abcG1*), has been associated with triazole resistance in A. fumigatus (22, 23, 38). Furthermore, deletion of *abcC* in both triazole-susceptible laboratory strains of A. fumigatus and a triazole-resistant clinical isolate has been shown to increase triazole susceptibility (22, 23, 38). As *cyp51A-* and *cyp51B*-mediated mechanisms alone do not adequately explain the high level of triazole resistance in this collection of clinical isolates, we sought to characterize the expression of *abcC* and its potential impact on triazole susceptibility. To accomplish this, each of the 26 clinical isolates in our collection was grown overnight at 37°C in *Aspergillus* minimal medium in biological triplicate, and RNA was extracted as previously described. RT-qPCR was then utilized to quantify the relative expression of *abcC* in each of the 21 triazole-resistant clinical isolates compared to the expression level observed among the 5 triazole-susceptible control isolates.

**FIG 4:**
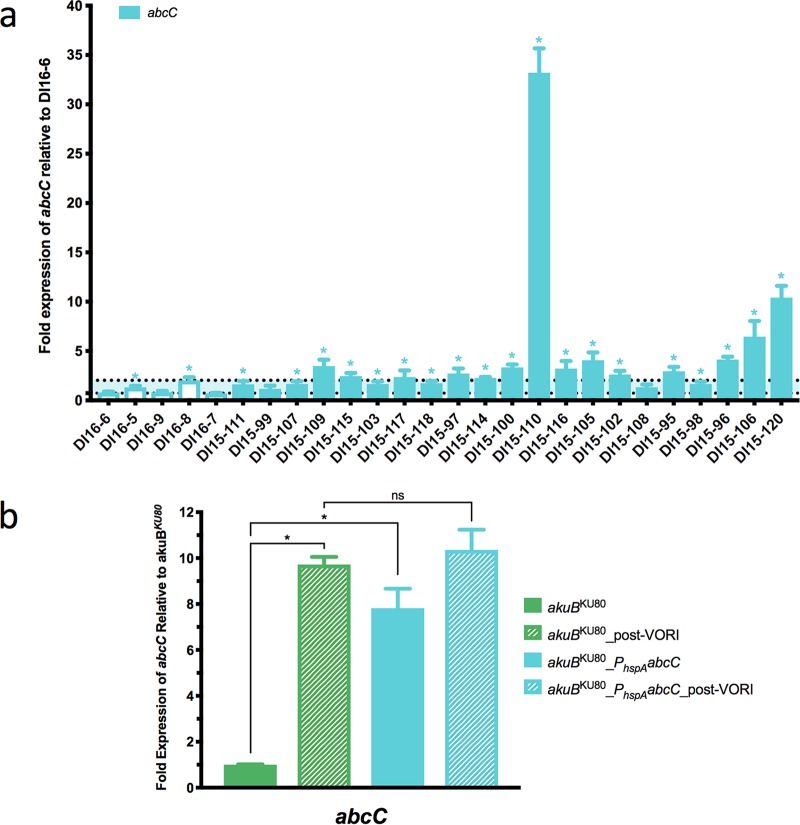
Relative expression of *abcC* in triazole-resistant clinical isolates and the constitutive overexpression strain *akuB*^KU80^_*P_hspA_abcC*. Triazole-susceptible isolates are shown as open bars; triazole-resistant isolates are shown as filled bars. Triazole-resistant isolates shown in order of escalating voriconazole MICs from left to right. Horizontal dotted lines denote highest and lowest expression levels observed among the 5 triazole-susceptible isolates. (a) Expression of *abcC* relative to triazole-susceptible isolate DI16-6. (b) The expression of *abcC* in strains *akuB*^KU80^ and *akuB*^KU80^_*P_hspA_abcC* was determined by RT-qPCR following growth in *Aspergillus* minimal medium at 37°C both with voriconazole (0.125 mg/liter) treatment for 6 h and without voriconazole treatment. The expression level for each sample is shown relative to that of the respective gene in *akuB*^KU80^ without voriconazole treatment. Comparisons with statistically significant (*P* < 0.05) results are noted with an asterisk, while those that are not are noted with “ns.”

The expression of *abcC* in all clinical isolates was compared to the triazole-susceptible isolate DI16-6. Among triazole-susceptible control isolates, the expression of *abcC* ranged from 0.7- to 2-fold that of isolate DI16-6 (Fig. 4a), with both isolates DI16-5 and DI16-8 exhibiting statistically greater *abcC* expression (1.3- and 2.0-fold, respectively) than DI16-6. The expression of *abcC* among triazole-resistant clinical isolates ranged from 1.2- to 33.2-fold that of the susceptible comparator isolate. Nineteen of the 21 triazole-resistant isolates were observed to have *abcC* expression that was statistically greater than the expression observed in the susceptible control isolate DI16-6, 14 of which exhibited *abcC* expression which also exceeded the highest expression level observed among triazole-susceptible isolates (2-fold). While the majority of these 14 isolates exhibited modestly elevated *abcC* expression (2.4- to 4.1-fold that of the comparator susceptible isolate), isolates DI15-110, DI15-120, and DI15-106 exhibited markedly higher levels of *abcC* expression (6.5-, 10.4-, and 33.2-fold, respectively). Notably, the pan-triazole-resistant isolate DI15-110, which exhibited the highest level of *abcC* expression, possesses no mutations in *cyp51A* and did not exhibit increased constitutive expression of either sterol-demethylase gene.

As previous studies associating increased triazole resistance with *abcC* have entirely relied upon deletion of the *abcC* gene among either clinical or laboratory isolates of A. fumigatus, we next sought to utilize our Cas9-RNP-mediated promoter replacement system to delineate the direct impact of constitutive overexpression of *abcC* on triazole MIC. To accomplish this, protoplasts of the triazole-susceptible laboratory strain *akuB*^KU80^ were transformed with Cas9-ribonucleoprotein (RNP) complexes, consisting of Cas9 protein and crRNA-trRNA duplexes targeting sequences immediately upstream of the *abcC* start codon, as well as the *P_hspA_abcC* transformation repair template, which contains both the *hspA* promoter and hygromycin resistance cassette flanked by 50 bases of microhomology targeting the *abcC* locus, yielding the strain *akuB*^KU80^_*P_hspA_abcC.* Subsequently, the relative expression of *abcC* in both *akuB*^KU80^_*P_hspA_abcC* and the parental strain *akuB*^KU80^ was assessed by RT-qPCR following growth in *Aspergillus* minimal medium at 37°C both with voriconazole treatment (0.125 mg/liter) for 6 h and without.

In the parental *akuB*^KU80^ strain, *abcC* expression was significantly (9.7-fold) higher following voriconazole treatment than was observed without voriconazole treatment (Fig. 4b). By comparison, the *akuB*^KU80^_*P_hspA_abcC* strain exhibited 7.8-fold- and 10.4-fold-higher *abcC* expression, under untreated and voriconazole-treated conditions, respectively, than the expression of *abcC* measured in the parental *akuB*^KU80^ under untreated conditions. *abcC* expression was not found to be significantly different between *akuB*^KU80^_*P_hspA_abcC* and the parental strain when both had been treated with voriconazole for 6 h. Surprisingly, even with an approximately 10-fold increase in the constitutive expression of *abcC*, the *akuB*^KU80^_*P_hspA_abcC* strain did not demonstrate any change in voriconazole susceptibility relative to the parental *akuB*^KU80^ strain (MIC, 0.25 mg/liter).

### Mutations in *hmg1* are common among triazole-resistant clinical isolates of A. fumigatus.

**FIG 5:**
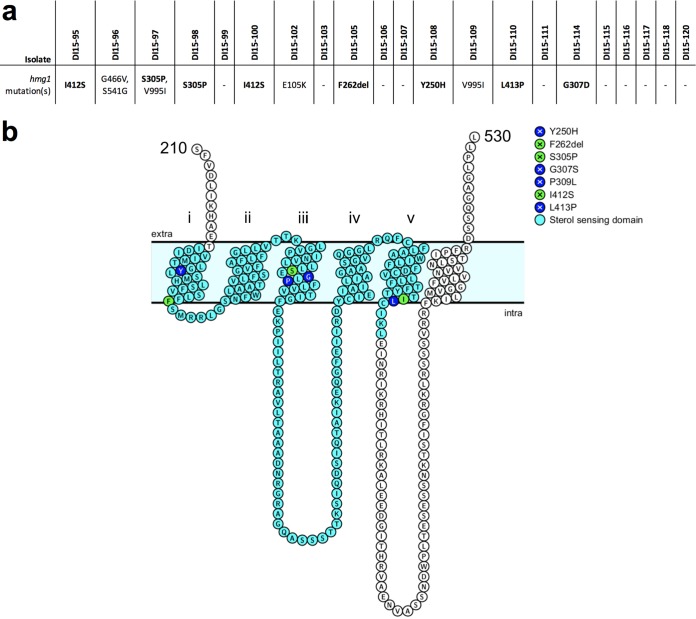
Mutations in *hmg1* observed in this collection of triazole-resistant clinical A. fumigatus isolates. (a) *hmg1* mutations observed in each clinical isolate relative to the Af293 reference genome, with mutations affecting residues predicted to reside within the conserved sterol-sensing domain shown in bold. (b) Schematic depicting the predicted transmembrane domains of A. fumigatus Hmg1. Residues in light blue comprise the predicted sterol-sensing domain, residues in dark blue represent those affected by *hmg1* mutations observed in this collection of clinical isolates, and residues shown in green represent those affected by *hmg1* mutations observed in this collection of clinical isolates and further characterized in this work.

Following the finding that the previously characterized mechanisms of triazole resistance did not explain the high level of triazole resistance observed in this collection of clinical A. fumigatus isolates, we sought to interrogate the previously generated whole-genome sequencing data to identify novel mechanisms of resistance. As the triazoles exert antifungal activity through the inhibition of biosynthesis of the predominant fungal membrane sterol ergosterol, initial whole-genome sequencing analysis targeted genes encoding proteins known to participate in ergosterol biosynthesis. In addition to the previously identified *cyp51A* mutations, mutations unique to triazole-resistant isolates were also observed in *erg3B* (Afu2g00320), *erg3C* (Afu8g01070), *erg4B* (Afu1g07140), *erg5* (Afu1g03950), and *erg6* (Afu4g03630). However, mutations occurred in these genes among only a small number of the clinical isolates (see Table S1 in the supplemental material). Intriguingly, 11 of the 21 triazole-resistant clinical isolates (52%) were found to possess mutations in the *hmg1* gene resulting in peptide sequence changes not observed among the triazole-susceptible control isolates (Fig. 5a). The A. fumigatus *hmg1* gene encodes a HMG-CoA reductase enzyme previously reported to be essential. HMG-CoA reductase catalyzes the first committed step in ergosterol biosynthesis and has previously been shown in other eukaryotic organisms to participate in the regulation of sterol biosynthesis through direct interactions between sterols and a conserved sterol-sensing domain (39–42). Of particular interest, eight of the 11 isolates with *hmg1* mutations were found to have mutations affecting residues predicted to reside within the conserved sterol-sensing domain of Hmg1 (Fig. 5b). We additionally accessed publicly available whole-genome sequencing data for 11 triazole-resistant clinical isolates (https://www.ebi.ac.uk/arrayexpress/experiments/E-SYBR-1/samples/) and found 5 to have mutations in *hmg1*, 2 of which alter residues in the predicted sterol-sensing domain (P309L and I412T). As mutations in *hmg1* were observed in a large proportion of clinical triazole-resistant isolates, and *hmg1* mutations have previously been observed among laboratory isolates of A. fumigatus grown in the presence of triazole antifungals and the closely related agricultural sterol-demethylase inhibitors, the observed mutations in *hmg1* were selected for further interrogation (43, 44).

10.1128/mBio.00437-19.2 TABLE S1 Mutations in other genes involved in ergosterol biosynthesis which are unique to triazole-resistant isolates. Download Table S1, DOCX file, 0.1 MB. Copyright © 2019 Rybak et al. 2019 Rybak et al. This content is distributed under the terms of the Creative Commons Attribution 4.0 International license.

### Mutations in *hmg1* confer resistance to the triazole class of antifungals.

To test the hypothesis that mutations in *hmg1* directly contribute to clinical triazole resistance in A. fumigatus, three mutations encoding amino acid substitutions or in-frame codon deletions in different transmembrane regions of the predicted *hmg1* sterol-sensing domain (F262del, S305P, and I412S) were selected for characterization (Fig. 5b). Each of these mutations and a wild-type control allele were directly introduced to the native *hmg1* locus of the triazole-susceptible laboratory strain *akuB*^KU80^ using a novel Cas9-RNP editing technique incorporating a split hygromycin B resistance marker (Fig. S1). Briefly, *hmg1* alleles including the open reading frame and approximately 500 downstream bases were amplified by PCR from DI15-98 (S305P), DI15-100 (I412S), DI15-105 (F262del), and *akuB*^KU80^ (wild-type control) using a 3′ primer which introduced the terminal 80 bases with homology to the 3′ end of the *hphR* hygromycin B resistance gene open reading frame. A partial hygromycin B resistance cassette, including the *gdpA* promoter and a truncated *hphR* gene lacking the terminal 40 bases, was then amplified by PCR from the pUCGH plasmid using primers that introduced approximately 70 bases of homology with the downstream region of *hmg1* (45). Protoplasts of *akuB*^KU80^ were then transformed with RNP complexes, consisting of Cas9 protein and crRNA-trRNA duplexes targeting sequences immediately upstream and approximately 500 bases downstream of the open reading frame of *hmg1*, and 2 μg of each portion of the split marker repair template (a single *hmg1* allele and the truncated hygromycin B resistance cassette), to produce *hmg1* mutant strains *akuB*^KU80^ *hmg1*^F262del^, *akuB*^KU80^ *hmg1*^S305P^, *akuB*^KU80^ *hmg1*^I412S^, and *akuB*^KU80^ *hmg1*^WT^.

10.1128/mBio.00437-19.1 FIG S1 Schematic of Cas9-ribonucleoprotein (RNP) editing technique incorporating a split hygromycin B resistance marker for creation of *hmg1* mutant strains. Download FIG S1, PDF file, 0.1 MB. Copyright © 2019 Rybak et al. 2019 Rybak et al. This content is distributed under the terms of the Creative Commons Attribution 4.0 International license.

**FIG 6:**
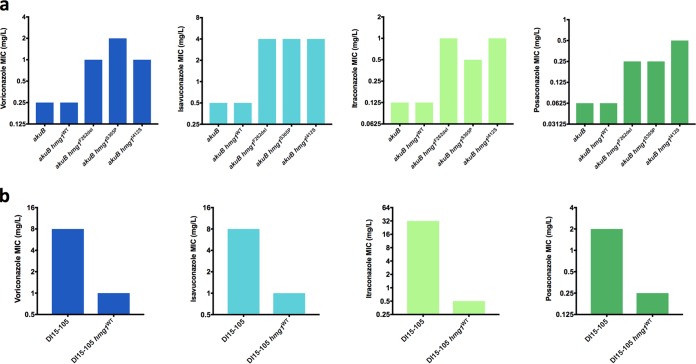
Impact of *hmg1* mutations on antifungal susceptibility. (a) Triazole MICs for *akuB*^KU80^ and derivative mutant *hmg1* strains. (b) Triazole MICs for DI15-105 and derivative *hmg1*^WT^ strain.

Antifungal susceptibility testing was subsequently performed for *akuB*^KU80^ and each of the derivative strains for voriconazole, isavuconazole, itraconazole, posaconazole, and amphotericin B according to the Clinical and Laboratory Standards Institute M38-A2 standards (Fig. 6a) (31). The parental *akuB*^KU80^ strain was susceptible to all agents with MICs of 0.25, 0.5, 0.125, 0.06, and 0.5 mg/liter for voriconazole, isavuconazole, itraconazole, posaconazole, and amphotericin B, respectively. The *akuB*^KU80^ *hmg1*^WT^ control strain exhibited MICs exactly matching those of the parental strain. Conversely, all strains harboring a clinically derived mutation in *hmg1* were found to exhibit a 4- to 8-fold increase in MICs for all tested triazoles, exceeding the CLSI reported epidemiologic cutoff values for at least one agent in each mutant strain. Notably, amphotericin B MICs were observed to decrease by 2-fold in both the *akuB*^KU80^ *hmg1*^S305P^ and *akuB*^KU80^ *hmg1*^F262del^ strains (0.5 versus 0.25 mg/liter) but not in *akuB*^KU80^ *hmg1*^I412S^.

In an effort to delineate the extent of *hmg1* mutation-mediated triazole resistance within a clinical isolate from our collection, the mutant *hmg1* allele in the pan-triazole-resistant clinical isolate DI15-105 was replaced with the wild-type *hmg1* allele from *akuB*^KU80^ using the same Cas9-RNP-mediated gene editing described above. The resultant DI15-105 *hmg1*^WT^ strain exhibited complete restoration of triazole susceptibility with an 8-fold or greater decrease in MIC for each triazole agent (Fig. 6b). Intriguingly, the amphotericin B MIC was observed to increase by 2-fold in DI15-105 *hmg1*^WT^ relative to DI15-105 (0.125 versus 0.25 mg/liter).

### Mutations in *hmg1* lead to accumulation of ergosterol precursors.

**FIG 7:**
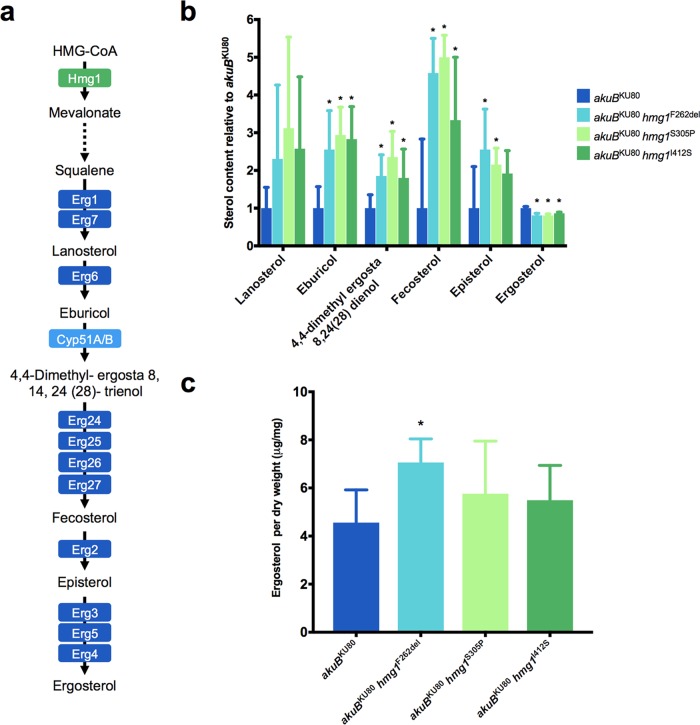
Relative distribution of sterols and total ergosterol content of *hmg1* mutant strains. (a) A. fumigatus ergosterol biosynthetic pathway. (b) Relative fold change of cellular sterols lanosterol, eburicol, 4,4-dimethylergosta-8,24(28)-dienol, fecosterol, episterol, and ergosterol in *hmg1* mutant strains compared to the parental *akuB*^KU80^ strain. (c) Total cellular ergosterol per dry weight for *hmg1* mutant strains and the parental *akuB*^KU80^ strain. Comparisons with the susceptible parental isolate *akuB*^KU80^ with statistically significant (*P* < 0.05) results are noted with an asterisk.

As the sterol-sensing domain of HMG-CoA reductase has previously been shown to interact with ergosterol precursors and participate in the negative regulation of HMG-CoA reductase activity in both human cells and the model fission yeast Schizosaccharomyces pombe, we hypothesized that the mutations identified in the sterol-sensing domain of A. fumigatus *hmg1* may be leading to dysregulation of the ergosterol biosynthesis pathway resulting in the observed triazole resistance (Fig. 7a) (39–42). To test this hypothesis, comprehensive sterol profiling including assessment of the relative distribution of cell sterols and the measurement of total ergosterol content per dry weight was performed on *akuB*^KU80^, *akuB*^KU80^ *hmg1*^F262del^, *akuB*^KU80^ *hmg1*^S305P^, *akuB*^KU80^ *hmg1*^I412S^, and *akuB*^KU80^ *hmg1*^WT^.

To assess the relative distribution of sterols among A. fumigatus strains harboring *hmg1* mutations, freshly harvested conidial stocks of each strain were grown in six biological replicates in RPMI medium supplemented with 0.2% glucose and buffered with MOPS (pH 7.0) for 24 h before cells were flash frozen with liquid nitrogen, dry weights were obtained, and nonsaponifiable lipids were extracted, derivatized, and analyzed by gas chromatography-mass spectrometry as described previously (46, 47).

In agreement with the hypothesis that mutations in *hmg1* precipitate dysregulation of the ergosterol biosynthetic pathway, the distribution of sterols was dramatically altered in all *hmg1* mutant strains relative to *akuB*^KU80^ (Fig. 7b). Most notably, a significant decrease in the relative proportion of ergosterol, along with corresponding increases in the proportions of several ergosterol precursors downstream of Hmg1, including eburicol, 4,4-dimethylergosta-8,24(28)-dienol, and fecosterol, were observed in all mutant *hmg1* strains. Additionally, the relative proportion of episterol was observed to be significantly elevated in *akuB*^KU80^ *hmg1*^F262del^ and *akuB*^KU80^ *hmg1*^S305P^. Importantly, no significant difference in the relative distribution of sterols was observed between *akuB*^KU80^ and *akuB*^KU80^ *hmg1*^WT^ (data not shown).

While the distribution of cellular sterols among A. fumigatus strains harboring *hmg1* mutations demonstrated a significant decrease in the relative proportion of cellular ergosterol, total ergosterol content per dry weight was not lower among any of the mutant *hmg1* strains (Fig. 7c). In fact, relative to the parental *akuB*^KU80^, *akuB*^KU80^ *hmg1*^F262del^ was observed to exhibit a significant increase in total ergosterol (4.56 versus 7.06 μg ergosterol/mg [dry weight]; *P* < 0.01). Taken together, these findings demonstrate *hmg1* mutations precipitate a relative increase in ergosterol precursors downstream of Hmg1, while maintaining or in some cases even increasing cellular ergosterol, leading to significantly altered cellular sterol profiles among mutant *hmg1* strains.

### Mutations in *hmg1* do not lead to increased expression of sterol-demethylase genes.

**FIG 8:**
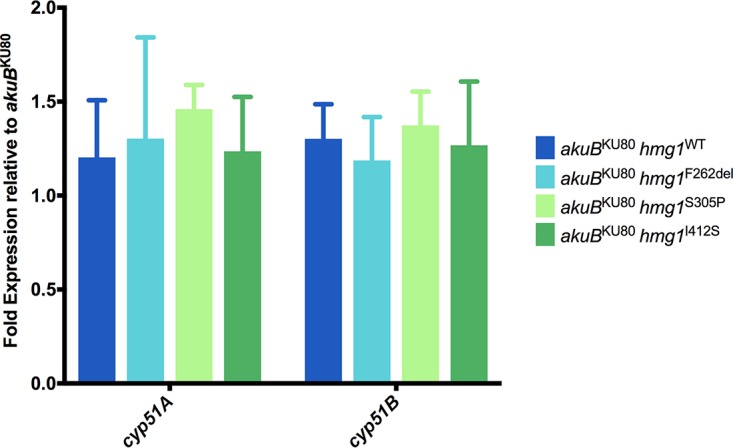
Expression of sterol-demethylase genes is not elevated among mutant *hmg1* strains. Fold expression of *cyp51A* and *cyp51B* in mutant *hmg1* strains relative to the parental *akuB*^KU80^ strain as determined by reverse transcription-quantitative PCR.

As our sterol profiling studies revealed significant accumulations of ergosterol precursors both upstream and downstream of sterol-demethylase, and we had previously observed that increased expression of either sterol-demethylase gene contributes to triazole resistance, we sought to investigate if the triazole resistance associated with mutations in *hmg1* was at least in part mediated by altered expression of sterol-demethylase genes. To accomplish this, conidial suspensions of *akuB*^KU80^, *akuB*^KU80^ *hmg1*^F262del^, *akuB*^KU80^ *hmg1*^S305P^, *akuB*^KU80^ *hmg1*^I412S^, and *akuB*^KU80^ *hmg1*^WT^ were grown in biological triplicates under the same conditions used for sterol profile analysis, and RNA was extracted from liquid nitrogen-pulverized samples, as previously described (35). RT-qPCR was then performed to assess expression of the sterol-demethylase genes *cyp51A* and *cyp51B* among mutant *hmg1* strains relative to the parental *akuB*^KU80^. No significant differences in expression of either sterol-demethylase gene were observed for any of the mutant *hmg1* strains or the *akuB*^KU80^ *hmg1*^WT^ control strain (Fig. 8). This finding demonstrated that while mutant *hmg1* strains are observed to have relatively greater proportions of ergosterol precursors, expression levels of both *cyp51A* and *cyp51B* remain unchanged. Thus, resistance in these strains is not due to increased expression of these genes.

## DISCUSSION

Resistance to the triazole class of antifungals represents a critical threat to the current treatment of invasive aspergillosis, a devastating disease state primarily afflicting the expanding populations of immunocompromised patients worldwide. While considerable research has been performed to interrogate the mechanisms by which pathogenic fungi such as *Aspergillus* become resistant to triazole antifungals, the overwhelming majority of resistance has been ascribed to three canonical paths that were originally described among pathogenic fungi decades ago: mutations in sterol-demethylase genes, overexpression of sterol-demethylase, or overexpression of efflux pump-encoding genes. However, these mechanisms alone or even in combination poorly explain a large proportion of the triazole resistance observed among clinical A. fumigatus isolates today.

In this work, we performed a comprehensive characterization of the direct contributions of previously identified mechanisms of triazole resistance in a collection of triazole-resistant clinical isolates of A. fumigatus. We observed that correction of *cyp51A* mutations in 10 pan-triazole-resistant clinical isolates restored wild-type susceptibility, to even a single triazole agent, in only 3 isolates. We then demonstrated that while constitutive overexpression of *cyp51A*, and to a lesser extent *cyp51B*, was common among isolates in this collection, increasing expression of either sterol-demethylase gene by as much as 18- to 36-fold in the triazole-susceptible *akuB*^KU80^ background had a limited effect on triazole MICs. Analogously, we then observed that while the expression of the ABC-type efflux pump-encoding gene *abcC* was higher among 14 of the triazole-resistant isolates in this collection, constitutive overexpression of this gene in the *akuB*^KU80^ background did not have any effect on voriconazole MIC. Upon revealing that the previously known mechanisms of clinical triazole resistance explain only a small fraction of the resistance observed in this collection, we then utilized whole-genome sequencing to identify a previously uncharacterized mechanism of resistance present among the majority of the isolates.

Here, we describe for the first time a novel and noncanonical mechanism of triazole antifungal resistance. Identified through whole-genome sequencing, mutations in the HMG-CoA reductase-encoding gene, *hmg1*, were found among the majority of triazole-resistant A. fumigatus clinical isolates in our collection. Of the observed mutations, most occurred in the conserved sterol-sensing domain of *hmg1*, which has been previously demonstrated to participate in the regulation of sterol biosynthesis in other eukaryotic organisms, including S. pombe. When three of these clinically derived *hmg1* mutations were independently introduced into a well-characterized triazole-susceptible strain of A. fumigatus using an optimized Cas9-mediated system to replace the native *hmg1* locus, resistance to all clinically available anti-*Aspergillus* triazole antifungals greatly increased. To confirm that mutations in *hmg1* are a novel mechanism of clinical triazole resistance, we employed the same system to correct a *hmg1* mutation identified in a pan-triazole-resistant clinical isolate of A. fumigatus and observed restoration of susceptibility to all triazoles.

As the sterol-sensing domain of HMG-CoA reductase has been shown to be essential to the regulation of sterol biosynthesis, we hypothesized that amino acid substitutions in this domain of Hmg1 may lead to impaired negative regulation of Hmg1 activity. In an effort to test this hypothesis, we performed comprehensive sterol profiling on the mutant *hmg1* strains. This analysis identified significant accumulations of ergosterol precursors, including eburicol, 4,4-dimethylergosta-8,24(28)-dienol, fecosterol, and episterol, while total cellular ergosterol content was seen to be sustained or, in the case of the *hmg1*^F262del^ mutation, significantly increased. However, this increase in ergosterol precursors did not result in a subsequent increase in sterol-demethylase expression levels in any of the mutant *hmg1* strains. Thus, *hmg1* mutation-mediated triazole resistance is not the result of increased sterol-demethylase gene expression.

Taken together, the results of this study identify mutations in the A. fumigatus HMG-CoA reductase gene, *hmg1*, as a novel and noncanonical genetic determinant of clinical triazole resistance, present among a large proportion of resistant clinical isolates. While the exact mechanism by which mutations in the sterol-sensing domain of *hmg1* impart clinical triazole resistance remains unknown at this time, it is tempting to speculate that the negative regulation of Hmg1 activity, which has been shown to be dependent on the sterol-sensing domain in other eukaryotic organisms, may be altered by residue substitutions in the sterol-sensing domain of A. fumigatus Hmg1.

## MATERIALS AND METHODS

### Isolates, media, and growth conditions used in this study.

Twenty-one previously characterized multitriazole-resistant clinical isolates of A. fumigatus, and five triazole-susceptible control clinical isolates of A. fumigatus, originating from the United States were obtained from the Fungus Testing Laboratory at the University of Texas in San Antonio. The laboratory strains *akuB*^KU80^ and Af293 were obtained from the Fungal Genetics Stock Center. All strains and clinical isolates used in this study were maintained on glucose minimal medium (GMM) agar at 37°C. Transformants were selected for using sorbitol (1.2 M)-supplemented GMM agar (SMM) containing 150 mg/liter of hygromycin. All conidia were harvested in sterile water from 3-day-old growth plates, and conidia were enumerated visually using a hemocytometer.

### Whole-genome sequencing.

For each isolate, genomic DNA was extracted using the Qiagen DNeasy Plant minikit as previously described (29). DNA concentrations were quantified using both the Qubit fluorometer and the NanoDrop spectrophotometer using the manufacturers’ protocols. Whole-genome libraries were prepared and sequenced at the University of Tennessee Health Science Center Molecular Resource Center. Bioinformatics services were provided by code4DNA. Sequence reads for each sample were aligned using the STAR sequence alignment tool (v2.5.0b) to the A. fumigatus reference sequence (version A_fumigatus_Af293_version_s03-m05-r05) which was downloaded from http://aspgd.org/. Duplicate alignments were marked using Picard (v1.119), and variants were called using Freebayes (v1.1.0) using the haploid population-based model. The Freebayes output vcf file was split into individual sample vcf files using bcftools (v1.3), and variants for each sample were annotated with snpEff (v4.2) using the A. fumigatus reference sequence annotations. Visualization of Hmg1 peptide sequence was performed using Protter (49).

### Construction of promoter replacement repair templates and Cas9-RNP for *cyp51A*, *cyp51B*, and *abcC*.

The plasmid pJMR2 was constructed by cloning the proximal 1 kb of the promoter sequence from the A. fumigatus heat shock protein-encoding gene, *hspA*, into the plasmid pCR-HygR (29). Then promoter replacement transformation repair templates for *cyp51A*, *cyp51B*, and *abcC* were created by PCR using primers (Table S2) which amplified both the hygromycin resistance cassette and the *hspA* promoter from the plasmid pJMR2, while also introducing approximately 40 bases of homology targeting sequences immediately upstream and downstream of the start codon for the respective gene of interest. PCR products were subsequently purified using the Gene Clean II kit (MP Biomedicals). Cas9-RNP complexes targeting sequences immediately upstream of the open reading frame of each gene of interest were assembled as previously described (29).

10.1128/mBio.00437-19.3 TABLE S2 Oligonucleotides used in this study. Download Table S2, DOCX file, 0.02 MB. Copyright © 2019 Rybak et al. 2019 Rybak et al. This content is distributed under the terms of the Creative Commons Attribution 4.0 International license.

### Assessment of *cyp51A*, *cyp51B*, and *abcC* expression by RT-qPCR.

For assessment of expression in clinical isolates, conidia from each isolate were allowed to germinate overnight in *Aspergillus* minimal medium incubated at 37°C on an orbital shaker at 250 rpm. For assessment of expression in constitutive overexpression strains constructed in the *akuB*^KU80^ background, conidia from each isolate were allowed to germinate overnight in *Aspergillus* minimal medium incubated at 37°C on an orbital shaker at 250 rpm and then transferred to fresh *Aspergillus* minimal medium either with voriconazole at 0.125 mg/liter (treated) or without voriconazole (untreated) for an additional 6 h at 37°C on an orbital shaker at 250 rpm. Then RNA was extracted from mature hyphae following liquid nitrogen crush as previously described (35). The RevertAid RT kit (Thermo Scientific) was utilized to synthesize cDNA. PCR master mix and SYBR were utilized to amplify A. fumigatus *cyp51A*, *cyp51B*, and *abcC* from cDNA by PCR per the manufacturer’s instructions. Table S2 lists the gene-specific primers used for PCR. Conditions used for PCR were as follows: AmpliTaq Gold activation at 95°C for 10 min, 40 cycles of denaturation at 95°C for 15 s, and annealing/extension at 60°C. The dissociation curve and threshold cycle (*C_T_*) was determined using the CFX96 real-time PCR system (Bio-Rad). Changes in gene expression among isolates were then calculated using the 2^−ΔΔ^*^CT^* method. All experiments were performed in triplicate from biological triplicates. As previously described, Δ*C_T_* values were used to calculate the standard error (35). Statistical analysis was performed using unpaired, two-tailed, Student’s *t* test in Prism 8 for Mac OS by GraphPad Software Inc. with significance set at 0.05 and degrees of freedom equal to 10.

### Construction of allele replacement repair templates and Cas9-RNP for the *cyp51A* locus.

The plasmid pCyp51A-HygR was created by cloning the proximal 1.5 kb of promoter sequence and open reading frame of the wild-type *cyp51A* allele from the genomic reference strain Af293, upstream of the hygromycin resistance cassette in the plasmid pCR-HygR, and then cloning the proximal 1.5 kb of the wild-type *cyp51A* terminator downstream of the hygromycin resistance cassette (29). Then, the *cyp51A*^WT^ transformation repair template was created by PCR using primers which amplified the wild-type *cyp51A* allele (including 500 bases of promoter sequence), the hygromycin resistance cassette, and 500 bases of the *cyp51A* terminator. Cas9-RNP complexes targeting sequences immediately upstream and approximately 500 bases downstream of the open reading frame of *cyp51A* were assembled as previously described (29). Target-specific guide sequences are listed in Table S2.

### Construction of allele replacement repair templates and Cas9-RNP for the *hmg1* locus.

Two-component repair templates consisting of a split hygromycin resistance marker (*hphR*) and *hmg1* alleles of interest were prepared by PCR. Briefly, *hmg1* alleles of interest including the open reading frame and approximately 500 downstream bases were amplified by PCR from DI15-98 (S305P), DI15-100 (I412S), DI15-105 (F262del), and *akuB*^KU80^ (wild-type control) using a 3′ primer which introduced the terminal 80 bases with homology to the 3′ end of the *hphR* hygromycin B resistance gene open reading frame. A partial hygromycin B resistance cassette, including the *gdpA* promoter and a truncated *hphR* gene lacking the terminal 40 bases, was then amplified by PCR from the pUCGH plasmid using primers that introduced approximately 70 bases of homology with the downstream region of *hmg1.* Primers used are listed in Table S2. PCR products were subsequently purified using the Gene Clean II kit (MP Biomedicals). Cas9-RNP complexes targeting sequences immediately upstream and approximately 500 bases downstream of the open reading frame of *hmg1* were assembled as previously described (29). Target-specific guide sequences are listed in Table S2.

### *Aspergillus* protoplast transformations.

Transformation of A. fumigatus protoplasts was performed as previously described with minor modifications (29). Approximately 2 μg of each portion of applicable transformation repair templates was then mixed with 200 μl of protoplasts, 26.5 μl of Cas9-RNP complexes, and 25 μl of polyethylene glycol (PEG)-CaCl_2_ buffer (60% [wt/vol] PEG 3350, 50 mM CaCl_2_·H_2_O, 450 mM Tris-HCl, pH 7.5) as previously described. Following an incubation on ice for approximately 1 h, 1.25 ml PEG-CaCl_2_ was added and the mixture was incubated for an additional 20 min at room temperature. The mixture was then diluted to a total volume of 3 ml with STC buffer (1.2 M sorbitol, 7.55 mM CaCl_2_·H_2_O, 10 mM Tris-HCl, pH 7.5) and plated on SMM agar plates. Plates were then incubated overnight prior to being overlaid with SMM top agar (GMM supplemented with 1.2 M sorbitol and 0.7% [wt/vol] agar) supplemented with hygromycin (final concentration of 150 g/ml), and the plates were incubated at 37°C for 3 days. Transformants were isolated to separate plates containing selective agar, genomic DNA was extracted, and successful CRISPR-Cas9 editing was confirmed by PCR screens using gene-specific primers and Sanger sequencing.

### Clinical antifungal susceptibility testing.

Susceptibilities for amphotericin B, voriconazole, isavuconazole, itraconazole, and posaconazole were determined for all isolates in accordance with CLSI M38-A2 methodology utilizing broth microdilution in RPMI (31). Each antifungal was obtained from the appropriate manufacturer. All agents were suspended in dimethyl sulfoxide (DMSO) for preparation of stock solutions.

### Comprehensive sterol profiling.

Fresh conidial suspensions of each strain to be studied were prepared in saline with Tween 80 from *Aspergillus* minimal medium agar plates. Conidia were grown in 25 ml RPMI supplemented with 0.2% glucose buffered to pH 7.0 with MOPS for 24 h in an orbital shaker at 180 rpm and 35°C. Cells were then flash-frozen using liquid nitrogen, dry weights were obtained, and alcoholic KOH was utilized to extract nonsaponifiable lipids. A vacuum centrifuge (Heto) was then used to dry samples, prior to derivatization by the addition of 200 μl of anhydrous pyridine (Sigma), 100 μl of *N*,*O*-bis(trimethylsilyl)trifluoroacetamide (BSTFA)–10% trimethylsilyl (TMS) (Sigma), and 2 h of heating at 80°C. Gas chromatography-mass spectrometry (GC-MS) (Thermo 1300 GC coupled to a Thermo ISQ mass spectrometer; Thermo Scientific) was then used to analyze and identify TMS-derivatized sterols. Known standards were referenced for fragmentation spectra and retention times. Sterol profiles for each isolate were then created using Xcalibur software (Thermo Scientific) to analyze GC-MS data (48). Statistical analysis of both sterol profiles and total ergosterol per dry weight was performed using GraphPad Prism 7. In all cases, 6 independent biological replicates were measured and included in analysis, and two-tailed unpaired *t* tests were performed in Prism 8 for Mac OS by GraphPad Software Inc. with significance set at 0.05 and degrees of freedom equal to 10.

### Data availability.

Whole-genome sequencing data files for the 26 Aspergillus fumigatus isolates have been deposited in NCBI SRA under the accession number PRJNA491253.
