# Supplementary figures and images for: Mutations in hmg1, Challenging the Paradigm of Clinical Triazole Resistance in Aspergillus fumigatus
Source: mBio. 2019 Apr 2;10(2):e00437-19. doi: 10.1128/mBio.00437-19 (PMC6445940; doi:10.1128/mBio.00437-19)

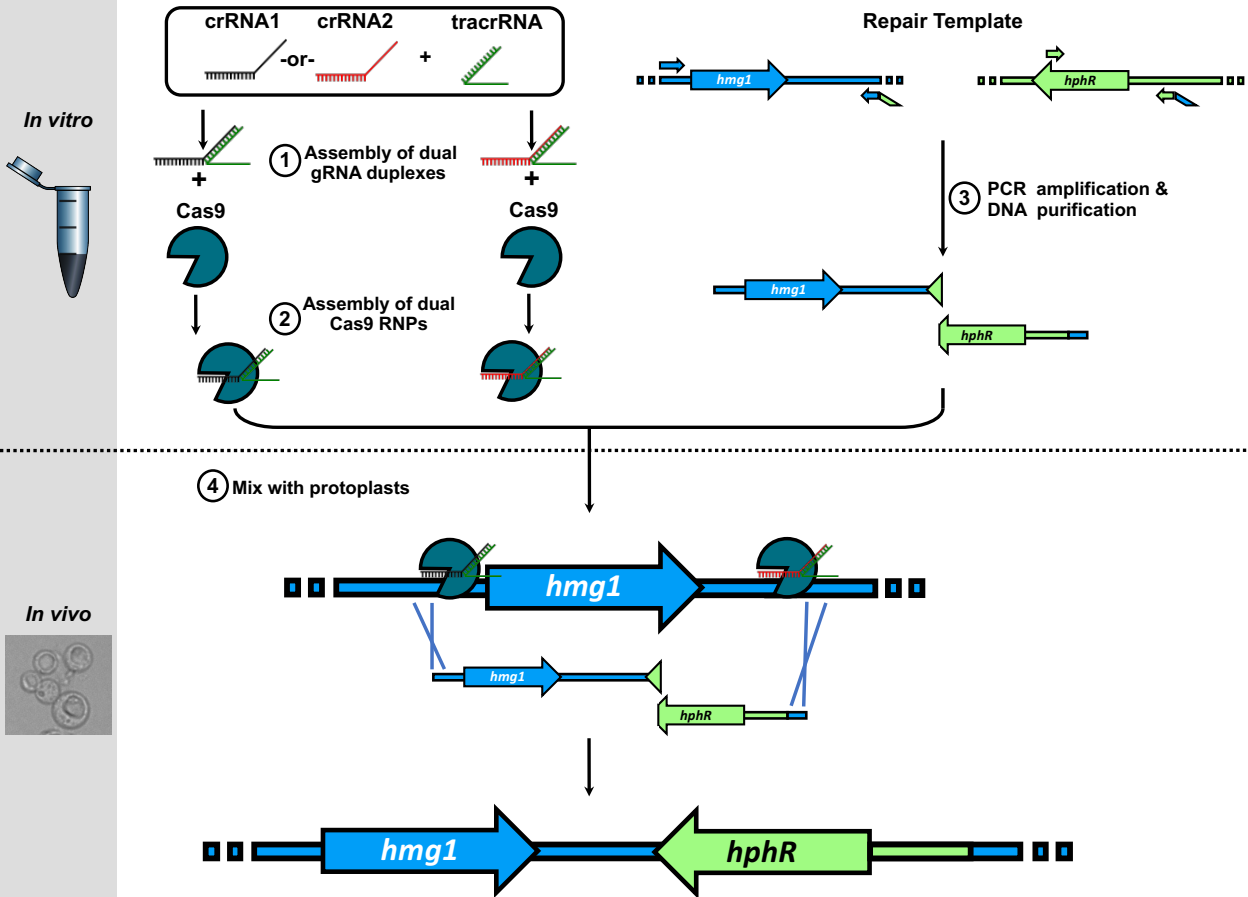

Supplement: FIG S1 [file mBio.00437-19-sf001.pdf]
