# Supplementary material for: Mutations in hmg1, Challenging the Paradigm of Clinical Triazole Resistance in Aspergillus fumigatus
Source: mBio. 2019 Apr 2;10(2):e00437-19. doi: 10.1128/mBio.00437-19 (PMC6445940; doi:10.1128/mBio.00437-19)
Supplement: TABLE S2 [file mBio.00437-19-st002.docx]

**Table S2.**

|  | **Gene** | **Primer Name** | **Sequence (5'- 3')** |
| --- | --- | --- | --- |
| **PCR Primers** | | | |
|  | *cyp51A* | *cyp51A* KI F CRISPR | GAATACTGGGCAGCGGGCTGGAGATACTATGGCTTTCATATGTTGCTCAGCGTCAGCATTCTGAAACACG |
|  | *cyp51A* | *cyp51A* KI R CRISPR | CCAGTAAGGTCTGAATAAGGGTTCAATACAGTCATTTATTAGGCCCTCGATTCGAGCTCCCAAATCTGTCCAG |
|  | *cyp51A* | *cyp51A* OE *hspA* F | CATTTCCCTCATCACTGCAACTCTAATCCTCGGGCTCACCCAGCTTGCATGCCTGCAGG |
|  | *cyp51A* | *cyp51A* OE *hspA* R | ACGGCCATGTAGGCCGTAAGCCATAGCATCGGCACCATGCTTGGTACCTGTGAAGAAGTG |
|  | *cyp51B* | *cyp51B* OE *hspA* F | CTACCTTTATTCCCTGCGACAGTAACTCGGTTGATCGATTTGAGCTTGCATGCCTGCAGG |
|  | *cyp51C* | *cyp51B* OE *hspA* R | TTGCAGATGCCGTCGAGAATGAACGCGATGAGACCCATGCTTGGTACCTGTGAAGAAGTG |
|  | *abcC* | *abcC* OE *hspA* F | GTTCGACTGTTTATGTGTATACCTATTGTGACGCAACCACTCAGCTTGCATGCCTGCAGG |
|  | *abcC* | *abcC* OE *hspA* R | GGGTTGATGTTAGGATTTATCGTCCCTAGAAGAGACATGCTTGGTACCTGTGAAGAAGTG |
|  | *hmg1* | *hmg1* F | GACAGGCGGCTTACACCGCCTCTCCTGCTCGCCATTTTGTCTATGATCACTGATGGCTACCTCTCTGATT |
|  | *hmg1* | *hmg1* split *hphR* R | TCTGGACCGATGGCTGTGTAGAAGTACTCGCCGATAGTGGAAACCGACGCCCCAGCACTCGTCCGAGGGCAAAGGAATAGGCTGCGTTACTCGGTCTTGG |
|  | *hphR* | *hphR* split F | CCACTATCGGCGAGTACTTCTACAC |
|  | *hphR* | *hphR* 3’UTR *hmg1* R | CCCACCTGGGATGAAGCAAAAGCGGGTACATAGATAGGTTTATGGGGACTTTGGGAAGTGAAATATGGGCGAGCTCCCAAATCTGTCCAG |
|  | *hmg1* | *hmg1* screen F | GGTGTCCGTGGTTCCCACC |
|  | *hphR* | *hphR* screen R | CAAATCGCCCGCAGAAGCG |
| **crRNA Guide Sequences** | | | |
|  | *cyp51A* | *cyp51A* 5’ crRNA | GGCTTTCATATGTTGCTCAG |
|  | *cyp51A* | *cyp51A* 3’ crRNA | TTCAAAGGATTTGGTGTGAT |
|  | *cyp51A* | *cyp51B* 5’ OE crRNA | CTCCCTGTGTCTCCTCGAAA |
|  | *cyp51B* | *cyp51B* 5’ OE crRNA | TATTCCCTGCGACAGTAACT |
|  | *abcC* | *abcC* 5’ OE crRNA | GACTGAGTGTCTCACTCAAT |
|  | *hmg1* | *hmg1* 5’ crRNA | ATTTTGTCTATGATAGACAA |
|  | *hmg1* | *hmg1* 3’ crRNA | CCAACGATTGCCAAAGGTCA |
| **RTqPCR Primers** | | | |
|  | *cyp51A* | *cyp51A* RT F e1 | CTTCTTTGCGTGCAGAGA |
|  | *cyp51A* | *cyp51A* RT R e2 | GGGGTCGTCAATGGACTA |
|  | *cyp51B* | *cyp51B* RT F e1 | CTTTTTCGACTGCCGCGC |
|  | *cyp51B* | *cyp51B* RT R e2 | AGGCGTAGTGAGTGGAGA |
|  | *abcC* | *abcC* RT F e1 | CCTGGAGAAGGTCTCAATGTCG |
|  | *abcC* | *abcC* RT R e2 | GGCCAGACGTAGGCTCATCC |
